# Chip-integrated polarization multiplexed metasurface for simultaneous generation of versatile terahertz vortices

**DOI:** 10.1515/nanoph-2025-0091

**Published:** 2025-04-22

**Authors:** Qianyun Zhang, Guibin Li, Liang Wu, Fan Yang, Zhen Yue, Chenglong Zheng, Yan Zhang, Li Li, Jianquan Yao

**Affiliations:** Key Laboratory of Opto-Electronics Information Technology (Tianjin University), School of Precision Instruments and Opto-Electronics Engineering, Tianjin University, No. 92 WeiJin Road, Tianjin, 300072, China; Department of Physics, Beijing Key Laboratory for Metamaterials and Devices, Key Laboratory of Terahertz Optoelectronics, and Beijing Advanced Innovation Center for Imaging Technology, Capital Normal University, Beijing, 100048, China; School of Physics, Harbin Institute of Technology, Harbin 150000, China; Department of Optoelectronic Information Science and Engineering, Jiangsu University, Zhenjiang 212013, China

**Keywords:** vortex beams, polarization multiplexed, metasurface, terahertz

## Abstract

Terahertz vortex beams, carrying orbital angular momentum (OAM), are quite desirable for enhancing data transmission capability in telecommunication. However, it faces fundamental and technical challenges in a single metasurface to simultaneously generate orthogonal basis vortices with linear polarization (*x*- and *y*-polarity) and circular polarization (left- and right-handed polarity) under the orthogonal polarized light incident. Here, we proposed a chip-integrated all-dielectric metasurface in the terahertz regime, to demonstrate the simultaneous generation of four-channel orthogonal polarized vortex beams at various topological charges under the *x*- and *y*-polarized light incident. The polarization multiplexed metasurface was designed only with a propagation phase strategy, consisting of polarization-maintaining and polarization-conversion meta-atoms. Simultaneous control of polarization and topological charges in vortex beams was realized by properly arranging birefringent meta-atom arrays to induce additional phases of *x*- and *y*-polarization as customized, showing more degrees of freedom for carrying information. The experimental results are in good agreement with the simulations. Such a metasurface approach provides complete polarization bases for further synthesis of diverse polarization vortices required for huge-capacity communication.

## 1 Introduction

Terahertz vortex beams carrying orbital angular momentum (OAM) possess the helical phase distribution and structured intensity profile, which attracted considerable attention due to their promising application in high-capacity telecommunications [1], [2], [3], particle manipulation [4], [5], quantum information processing [6], [7], [8], and super-resolution microscopic imaging [9]. More interestingly, the combination of two vortex fields at various topological charges can produce more diverse vortices by the superposition states of photonic OAM, as well as arbitrary vortices via the superposition of multiple OAM [10], [11]. This can provide effective tools for high-speed kinematic sensing [12] and spin object detection [13]. Traditional methods to generate vortex beams include the spiral phase plate [14], mode conversion [15], and computational holography [16]. The further superposition of vortices at various OAMs can be performed by interferometers [6], [8], [11], [17]. The traditional methods suffer from a limited number of topological charges, inflexible polarization manipulation, bulky optical systems with complex algorithms, and degraded performance due to optical misalignment [14], [15], [16], [17].

To tackle these challenges, metasurface approaches were developed instead of those traditional methods to generate diverse vortices and manipulate OAM superposition. Metasurface possess the unprecedented capability of electromagnetic manipulation at the subwavelength scale [18], [19], [20], [21], [22], [23], [24] and have been used to develop various multifunctional metadevice, including multiple focal points metalens [21], [22], [23], bandgap engineering [25], and multitarget and multiwavelength metasurface [26], and polarization manipulation metasurface [27], [28], [29], [30], [31], [32]. The polarization control and multifunctional integration of metasurface have played an important role in optics. The linearly polarized OAM superpositions between two vortices [33], as well as between the spherical and vortex waves [34], were performed by a spiral phase metalens in one same spatial channel. Under the circular polarization incidence, a single linear-polarized OAM superposition was presented by a spin-decoupling scheme with the combination of both geometric and propagation phases [35]. The geometric metasurface with complex phase integration was proposed to manipulate linearly polarized OAM superpositions of visible vortices, with intercoupling topological charges in multiple optical channels [36]. Previous studies on metasurfaces showed the feasibility of the generation and superposition of vortex beams, but with complex design scenarios and intercoupling effects among multiple vortices fields [34], [35], as well as without the capability of orthogonal polarization multiplexing output.

In the paper, a chip-integrated polarization multiplexed metasurfaces approach is exploited to generate diverse vortex beams, particularly to manipulate OAM superposition states with a new method of controlling the phase difference between two fundamental vortices. Experimentally, an all-dielectric metasurface in terahertz regime was prepared to demonstrate the simultaneous generation of four-channel orthogonal polarized vortex beams at various topological charges. The versatile metasurface was designed only with propagation phase strategy. The required phases were introduced by adjusting the size of silicon ellipse cylinders. The metasurface samples were fabricated on all-silicon geometries meta-atoms. By control of the phase response of polarization-maintaining and polarization-conversion meta-atoms, the linear co- and cross-polarization of fundamental vortex states were performed in the 1 and 2 channels; meanwhile, the left- and right-handed circular polarization of superposition vortex states were produced in the 3 and 4 channels. Furthermore, orthogonal linear and circular polarization vortex beams at various topological charges were measured in four spatial-decoupling channels under a linear polarization incidence (*x*- or *y*-polarity). Simultaneous control of polarization and topological charges in vortex beams was realized by properly arranging birefringent meta-atoms arrays to induce additional phases of *x*- and *y*-polarization as customized. The experimental results are in good agreement with the simulations.

## 2 Design and method

### 2.1 Scheme of four-channel vortex generation

**Figure 1:**
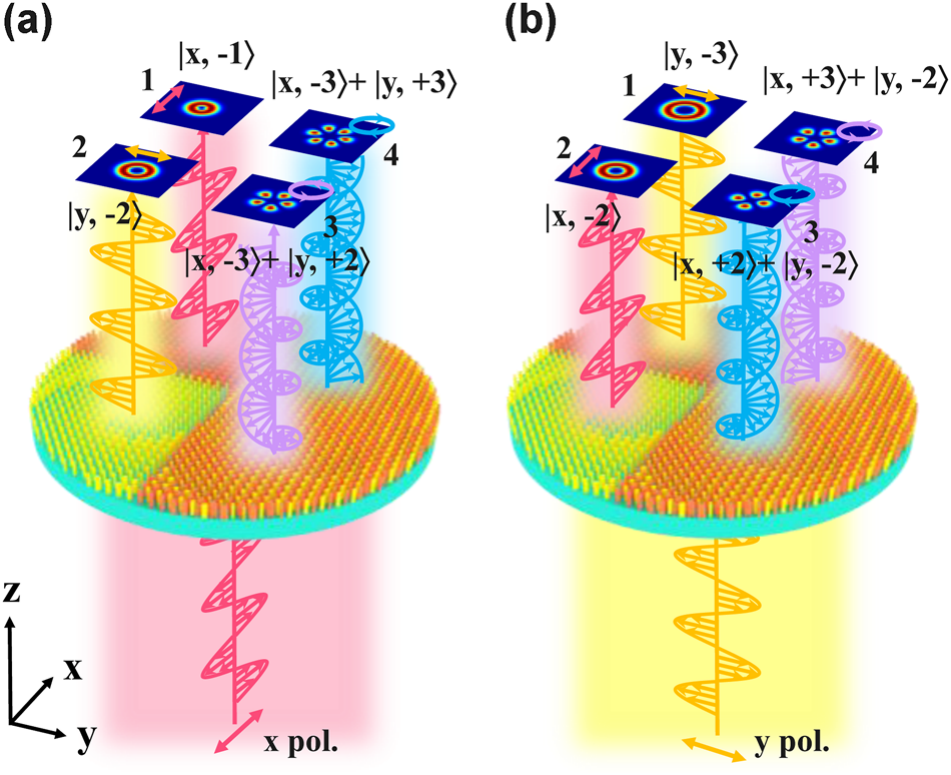
The schematic diagram of four-channel orthogonal polarized vortex beams at various topological charges. (a) Under *x*-polarization incidence, the fundamental vortex beam with |*x*, −1⟩ and |*y*, −2⟩ were performed in the 1 and 2 channels; meanwhile, the left- and right-handed circular polarization of superposition vortex states with |*x*, −3⟩ + |*y*, +2⟩ and |*x*, −3⟩ + |*y*, +3⟩ were produced in the 3 and 4 channels. (b) Under *y*-polarization incidence, the fundamental vortex beam |*y*, −3⟩ and |*x*, −2⟩ were generated in the 1 and 2 channels, and the left- and right-handed circular polarization of superposition vortex states with |*x*, +2⟩ + |*y*, −2⟩ and |*x*, +3⟩ + |*y*, −2⟩ were produced in the 3 and 4 channels.

Schematic of the proposed polarization multiplexed metasurface for generating the four-channel orthogonal polarized vortex beams at various topological charges under the orthogonal polarized light incident is illustrated in Figure 1. The metasurface is composed of two geometries of meta-atoms and is divided into four subarrays. The two meta-atoms consist of cross-ellipse silicon cylinders marked by yellow and orange, respectively. For the first structure (orange), two long axes along the *x*- and *y*-axes maintain the polarization state of the incident beam, under the linear polarization incidence. The other structure (yellow) with two long axes of the cross-ellipse cylinder along the *x*- and *y*-axes at an angle of 45 or −45° can realize efficient cross-polarization conversion when the linear polarization incidence is incident. The subarrays in the first channel of the metasurface are constituted by the meta-atoms with the function of polarization-maintaining; in the second channel, all the meta-atoms are achieving polarization-converting; two geometries of meta-atoms with different functions are interlaced and differ by half a period (*p*/2) in the third and fourth channel. Due to such flexible design, the metasurface can simultaneously generate four-channel orthogonal polarized vortex beams at various topological charges. As shown in Figure 1(a), under *x*-polarization incidence, the fundamental vortex beam with different polarization and topological charges, |*x*, −1⟩ and |*y*, −2⟩, were performed in the 1 and 2 channels; meanwhile, superposition vortex states of the right- and left-handed circular polarization with |*x*, −3⟩ + |*y*, +2⟩ and |*x*, −3⟩ + |*y*, +3⟩ were produced in the 3 and 4 channels. In Figure 1(b), under *y*-polarization incidence, the vortex beam |*y*, −3⟩ and |*x*, −2⟩ were generated in the 1 and 2 channels, and the superposition vortex states of left- and right-handed circular polarization with |*x*, +2⟩ + |*y*, −2⟩ and |*x*, +3⟩ + |*y*, −2⟩ were produced in the 3 and 4 channels.

To simultaneously generate orthogonally polarized vortex beams at various topological charges in four channels under the orthogonal polarized light incident, we first describe the physical mechanism for the generation of an orthogonally polarized state under the *x*- and *y*-polarized light incident. The birefringent meta-atoms are induced by the shape characteristics different from conventional optical devices, which are realized by the anisotropy of crystal materials [37]. When the incidence polarization direction is consistent with the axis of symmetry of the anisotropic atom, the transmitted polarization state is the same as the incident, and the incident polarization beam is called the intrinsic polarization (copolarization). The polarization conversion will result in the projection of the intrinsic polarization when the incidence polarization direction has an angle with the axis of symmetry of the meta-atom. In our work, the birefringent effect of meta-atoms was used, when positioned horizontally along the *x*-axis or at a 45° angle, enabling independent polarization control of orthogonal linearly polarized light through adjustments to its size. Compared to the hybrid phase approach [32], the single-phase method offers fewer degrees of freedom, which simplifies the design process and enhances processing accuracy. We assumed that an *x*- or *y*-polarization was incident. Due to our metasurface consisting of two different geometries meta-atoms, the transmitted electric field can be described as:

(1) $$\left[\begin{array}{c} E_{x}^{o}\\ E_{y}^{o} \end{array}\right]=T\cdot \left[\begin{array}{c} E_{x}^{i}\\ E_{y}^{i} \end{array}\right]=\left[\begin{array}{cc} T_{xx} & T_{xy}\\ T_{yx} & T_{yy} \end{array}\right]\cdot \left[\begin{array}{c} E_{x}^{i}\\ E_{y}^{i} \end{array}\right]$$

where $\left[\begin{array}{c} E_{x}^{o}\\ E_{y}^{o} \end{array}\right]$ and $\left[\begin{array}{c} E_{x}^{i}\\ E_{y}^{i} \end{array}\right]$ represent the transmitted and the incident electric field, respectively. **T** is the transfer Jones matrix and consists of transmission coefficients $T_{ij}=t_{ij}{\text{e}}^{\text{i}\varphi_{ij}}$ , where *T*_*ij*_, *t*_*ij*_, and *φ*_*ij*_ indicate the transmission coefficient, amplitude, and phase of the transmitted *i*-polarized component corresponding to the *j*-polarization incidence.

In the first channel of the metasurface, all the meta-atoms generate copolarization for the incident polarization. The transmission Jones matrix of the metasurface is $T_{co}=\left[\begin{array}{cc} T_{xx} & 0\\ 0 & T_{yy} \end{array}\right]$ , when an *x*-polarization light is incident, the transmitted field $E_{x}^{o}=t_{xx}{\text{e}}^{\text{i}\varphi_{xx}}\cdot E_{x}^{i}$ only has an *x*-polarization component. When a *y*-polarization is incident, the output field is also intrinsically polarized.

In the second channel, all the meta-atoms convert the incident polarization into cross-polarization, and the unconverted part can be neglected when the conversion efficiency is high enough. The long axis of the cross-ellipse cylinder of the meta-atoms is oriented at an angle of 45 or −45° between the *x*- and *y*-axes. The transmission matrix $T=T_{cross}=\left[\begin{array}{cc} 0 & T_{yx}\\ T_{yx} & 0 \end{array}\right]$ , where the $t_{xy}{\text{e}}^{\text{i}\varphi_{xy}}=t_{yx}{\text{e}}^{\text{i}\varphi_{yx}}$ are defined as $t_{\text{cross}}{\text{e}}^{\text{i}\varphi_{\text{cross}}}$ . When an *x*-polarization light is incident, the transmitted electric field is $E_{y}^{o}=t_{yx}{\text{e}}^{\text{i}\varphi_{yx}}\cdot E_{x}^{i}$ . The output field is *y*-polarization. In the same way, we can also achieve *x*-polarization transmission under *y*-polarization incidence.

In the third and fourth channels, the polarization-maintaining and polarization-conversion meta-atoms are staggered arrangements in space. The transmission matrix of the metasurface is $T=T_{\mathrm{co}}+T_{\mathrm{cross}}=\left[\begin{array}{cc} T_{xx} & 0\\ 0 & T_{yy} \end{array}\right]+\left[\begin{array}{cc} 0 & T_{yx}\\ T_{yx} & 0 \end{array}\right]$ . When an *x*-polarization light is incident, the transmitted field is $E^{o}=E_{x}^{o}+E_{y}^{o}=t_{xx}{\text{e}}^{\text{i}\varphi_{xx}}\cdot E_{x}^{i}+t_{yx}{\text{e}}^{\text{i}\varphi_{yx}}\cdot E_{x}^{i}$ . The transmission amplitude assumed unity; it can be found that the output polarization is determined by the phase difference between the transmitted *x*- and *y*-polarization components. We define the phase difference as *δ* = *φ*_*xx*_−*φ*_*yx*_. Under the *x*-polarization incidence, we design *δ* = *π*/2 in third channel and *δ* = −*π*/2 in fourth channel, and the RCP (right-hand circular polarization) and LCP (left-hand circular polarization) are generated, respectively. Under the *y*-polarization is incident, the *δ* = −*π*/2 and *δ* = *π*/2 in the third and fourth channels, LCP and RCP light are produced, respectively.

### 2.2 Design of the meta-atoms

**Figure 2:**
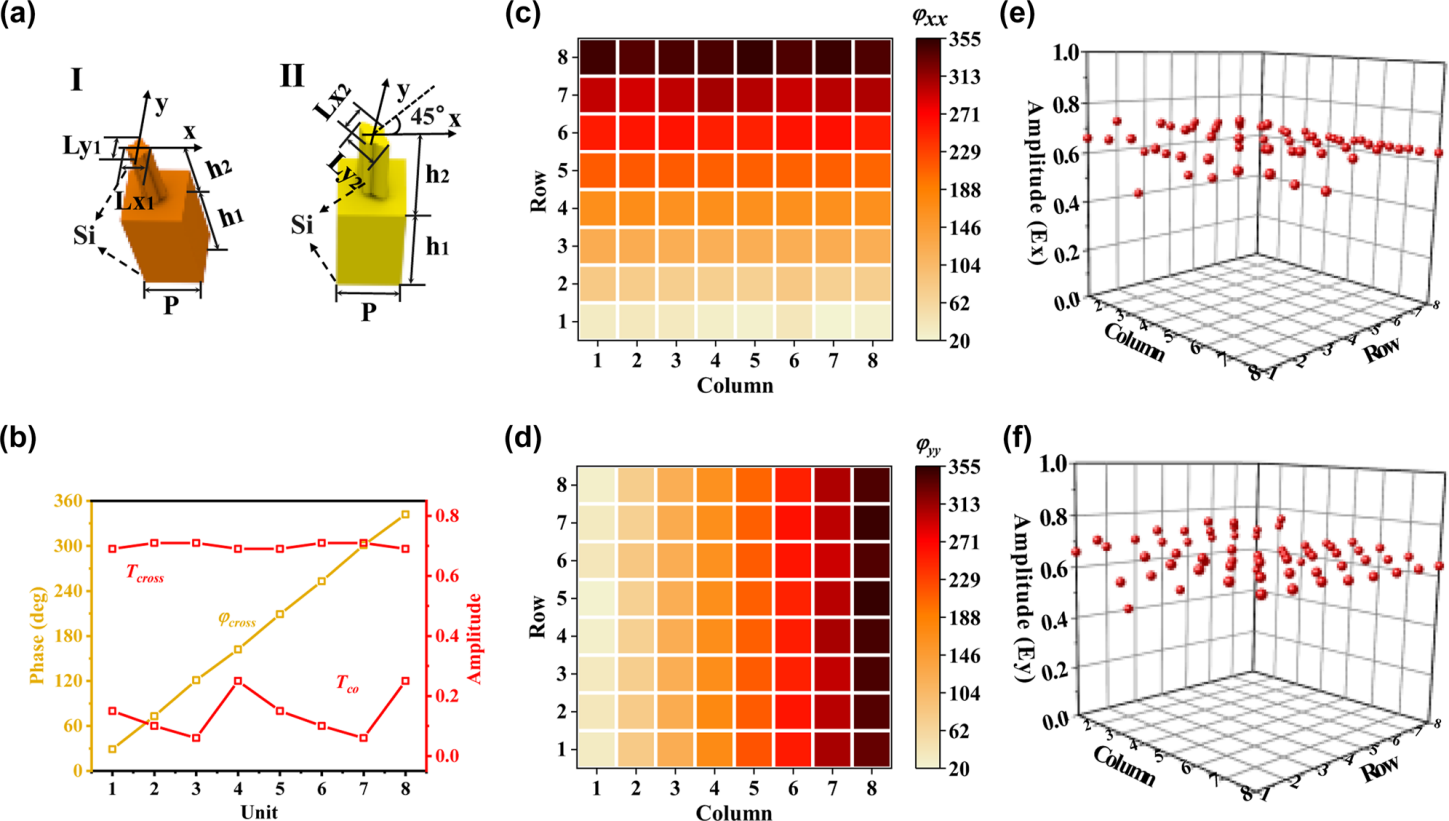
Database of the selected two geometric meta-atoms. (a) Schematic of the two meta-atoms. (b) Transmission amplitudes and phase shifts of meta-atom II as a function of Lx2 and Ly2. (c–f) Phase shifts and transmission amplitudes of meta-atom I with different parameters Lx1 and Ly1.

To achieve the mentioned above functions by two different geometries meta-atoms in Figure 2(a), the Finite-Integration-Technique (FIT) algorithm is used to build parameter libraries and select the needed meta-atoms. The periodic boundary is used along the *x* and *y* directions, and the open boundary is used along the *z* direction. The *x*-polarized (*y*-polarized) plan wave is the wave source. The probe is placed at a distance of two wavelengths from the meta-atom. We adopted high-resistance Si (silicon, *ρ* > 5,000 Ω m and *ε* = 11.9) to prepare the meta-atoms due to its excellent characteristics of high transmittance and low loss in the terahertz regime, and the dielectric material Si was indicated in the structure. The meta-atoms (I) and (II) have the same period of *p* = 150 μm, a uniform substrate thickness of h1 = 300 μm, and an equal height of h2 = 200 μm. The minor axes of the cross-ellipse cylinders of both meta-atoms are 30 μm. The long axes (Lx1, Ly1, Lx2, and Ly2) of the two meta-atoms were scanned as variables, where the values of Lx1 and Ly1 ranged from 30 to 130 μm with intervals of 2 μm, while Lx2 and Ly2 ranged from 30 to 60 μm and 60–90 μm, respectively. The response of the transmission amplitude and phase shift at 1.3 THz under the illumination of *x*- and *y*-polarization can be seen in Figure S1 (Supporting Information). Firstly, we selected 8 meta-atoms II, as shown in Figure 2(b), whose phase delay covers 360° with a step of 45°, the cross-polarization amplitude of all elements is above 0.6, and the copolarization amplitude is below 0.3. Therefore, the selected meta-atoms can achieve flexible phase manipulation and efficient polarization conversion. We selected 64 meta-atoms I from the scanned parameter library, as shown in Figure 2(c)–(f). The numbers from 1 to 8 indicate the phase shift from 0° to 315° with an interval of 45°. We can observe that the copolarization phase response of 8 meta-atoms in a certain column (row) for the *x* (*y*) polarization is the same value, but for the *y* (*x*) polarization, it covers 0°–315°. This means the selected 64 meta-atoms can perform independent copolarization phase control for the *x* and *y* polarization. The copolarization amplitude of the selected meta-atoms is almost all around 0.6 under a linear polarization incidence (*x*- or *y*-polarity). With the combination of the two geometrics of meta-atoms, the cross- and copolarization can be controlled independently and simultaneously.

## 3 Results and discussion

### 3.1 Four-channel orthogonal polarization generation

**Figure 3:**
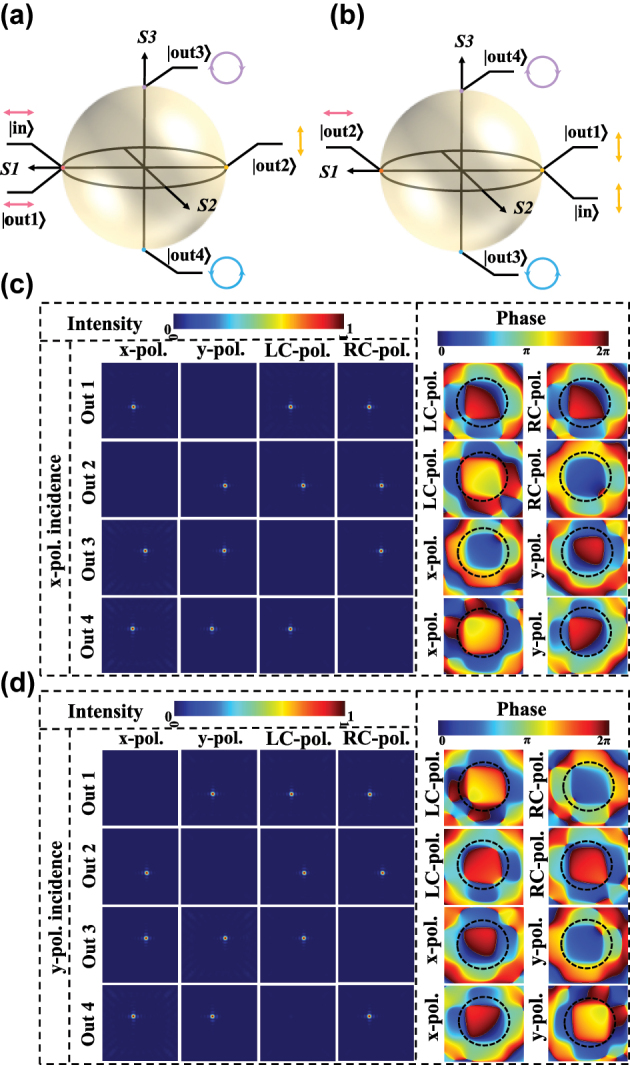
Polarization components analysis of simulation results for *x*- and *y*-polarization incidence. (a) and (b) Illustrations of two orthogonal polarization basis generations on the Poincaré sphere with different incident polarization. (c) and (d) Electric field intensity and phase distributions of the transmitted polarization components in four channels under *x*- and *y*-polarization incidence.

In order to simultaneously generate four-channel orthogonal polarized vortex beams at various topological charges, we first studied the generation of two orthogonal polarization bases in four-channel. The Poincaré spheres are introduced to illustrate the incident and transmitted polarization [31], in which they are denoted |in⟩ and |out⟩. As shown in Figure 3(a) and (b), when *x*- and *y*-polarization is incident, the orthogonal basis with linear polarization (*x*- and *y*-polarity) and circular polarization (RCP and LCP) are transmitted in four different channels, respectively. The meta-atoms are staggered arrangements at the metasurface to make full use of the space. The normal and alternate meta-atoms differ by half a period, and the different numbers represent different channels. The designed phase distributions of the four channels are as follows.

In the first channel, all meta-atoms are of geometry I, and the transmitted copolarizations light obtain phase delays *φ*_co_. The phase distributions of the meta-atoms are described as:

(2) $$\varphi_{\text{co}1}=\frac{2\pi }{\lambda }\left(\sqrt{{\left(x-\xi \right)}^{2}+{\left(y-\xi \right)}^{2}+f^{2}}-f\right)$$

However, for the second channel, all meta-atoms are of geometry II, which means that cross-polarization conversion is achieved. The phase response is

(3) $$\varphi_{\text{cross}2}=\frac{2\pi }{\lambda }\left(\sqrt{{\left(x+\xi \right)}^{2}+{\left(y-\xi \right)}^{2}+f^{2}}-f\right)$$

In addition to only producing copolarization or cross-polarization of the incident polarization in the transmitted field, we hope to generate both polarizations in one channel under the *x*- or *y*-polarization incidence. In this way, other polarizations can be derived by superimposing the two transmitted waves with a certain phase difference. Hence, the two geometrics (I and II) meta-atoms are arranged in the metasurface’s third and fourth channels. The phase distribution of these two channels is as follows:

$$\;\varphi_{\text{co}3}=\frac{2\pi }{\lambda }\left(\sqrt{{\left(x+\xi \right)}^{2}+{\left(y+\xi \right)}^{2}+f^{2}}-f\right)+\frac{\pi }{2}$$

(4) $$\varphi_{\text{cross}3}=\frac{2\pi }{\lambda }\left(\sqrt{{\left(x+\xi +\frac{P}{2}\right)}^{2}+{\left(y+\xi -\frac{P}{2}\right)}^{2}+f^{2}}-f\right)$$

$$\;\varphi_{\text{co}4}=\frac{2\pi }{\lambda }\left(\sqrt{{\left(x-\xi \right)}^{2}+{\left(y+\xi \right)}^{2}+f^{2}}-f\right)+\frac{\pi }{2}$$

(5) $$\varphi_{\text{cross}4}=\frac{2\pi }{\lambda }\left(\sqrt{{\left(x-\xi +\frac{P}{2}\right)}^{2}+{\left(y+\xi -\frac{P}{2}\right)}^{2}+f^{2}}-f\right)$$

The focal length *f* of the metasurface with four channels is designed to be 6,000 µm. The factor *ξ* is 2,550 μm, which is determined by the spatial coordinates of the focal point and the defined range for optimal focus. The working frequency is 1.3 THz. The phase *φ*_co_ is the copolarization phase shifts, while the *φ*_cross_ represents the cross-polarization response when *x*- or *y*-polarization is incident. The meta-atoms in the metasurface were properly arranged based on the above phase distribution in four-channel.

The simulation results for the electric field intensity and phase distribution of the transmission field polarization components can be seen in Figure 3(c) and (d) in the four channels, where the values are normalized. These four transmission channels can generate different polarization states independently under the incidence of *x*- and *y*-polarization. In channel 1, we can observe that the *y*-polarization electric field component is zero, the phase shift and field intensity of LCP and RCP components are equal at the same time, and the *x*-polarization component exists. Therefore, the *x*-polarization state is transmitted when the *x*-polarization is incident. Under the *y*-polarization incidence, the intensity of LCP and RCP is identical, but the phase delay differs by *π*; hence, the *y*-polarization is transmitted, and the *x*-polarization is absent. We find that the simulation results are in good agreement with the design phase expectations. The second row of Figure 3(c) and (d) demonstrates the output of channel 2. When the *x*-polarization is incident, the *y*-polarization is produced, which can be proved by the transmission of LCP and RCP components with the same intensity and *π* phase difference. While the *x*-polarization is generated under the *y*-polarization incidence, we can also find it from the uniform intensity value and phase of LCP and RCP components in the output field. In the third channel, the polarization-maintaining and polarization-conversion meta-atoms are interleaved. We observe that the simulated consequence is in coincidence with the designed phase distribution. When *x*-polarization is incident, the phase of the *x*-polarization component is ahead by *π*/2 of the *y*; therefore, the RCP is generated. The LCP light is produced under *y*-polarization illumination, which was demonstrated with the *y*-polarization component ahead of the *x*-polarization component phase of *π*/2. However, in channel 4, the meta-atoms phase of polarization-maintaining is behind *π*/2 of polarization-conversion. As illustrated in the last row of Figure 3(c) and (d), the LCP is generated under the *x*-polarization incidence; when the *y*-polarization is incident, the RCP output. The generated two orthogonal polarizations can act as complete polarization bases for further synthesis of versatile polarization.

**Figure 4:**
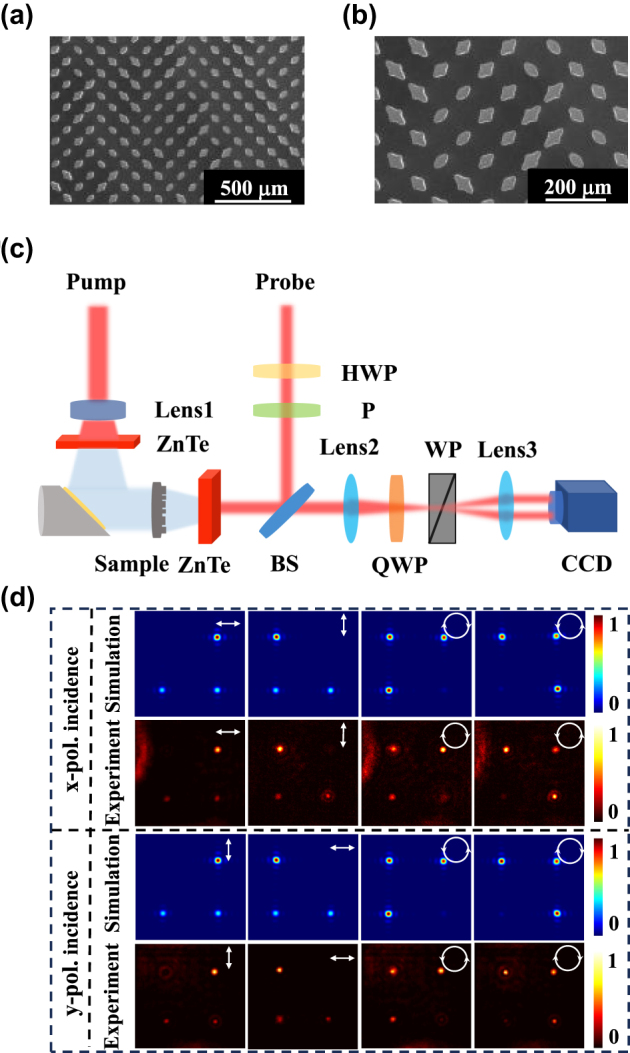
Experimental and simulated results for the generation of focused versatile polarization states. (a–b) SEM image of the fabricated versatile polarization states generator. (c) Schematic diagram of the THz holographic imaging system. (d) Transmitted field intensity profiles at the focal plane for different polarization bases.

The metasurface generation of two orthogonal polarization bases was fabricated using 500 µm thick high-resistance silicon through inductively coupled plasma (ICP) etching technology, and the process details are in Section S2 (Supporting Information). The sample effective part is a square of 1.2 cm in side length with 80 meta-atoms in the *x*- and *y*-axis directions. The sample scanning electron microscope (SEM) images are shown in Figure 4(a) and (b). It can be seen that the shape of the meta-atom is clear, which indicates the process accuracy is high. The THz holographic imaging system is used to characterize the sample, as shown in Figure 4(c). The working principle of the system is in Section S3 (Supporting Information). When the *x*- and *y*-polarization illuminates the sample at normal vertical incidence, we measure the transmitted field intensity profiles at the different polarization bases on the focal plane, and the corresponding simulation results are given simultaneously, as shown in Figure 4(d). The electric field intensity marked with different white polarization symbols represents different polarization-transmitted fields that we have detected. It can be observed that the *x*-polarization light, *y*-polarization, RCP, and LCP are independently generated in the four different channels when the *x*-polarization or *y*-polarization is incident. The polarization conversion efficiency was also calculated by the equation as follows:

(6) $$\eta =\frac{{\left|E_{\text{obj}}\right|}^{2}}{{\left|E_{\text{obj}}\right|}^{2}+{\left|E_{\text{obj }-\text{ cross}}\right|}^{2}}$$

**E**_obj_ is the transmitted objective wave, and **E**_obj−cross_ represents the cross-polarized wave of the objective. The conversion efficiency of the four-channel simulation result can reach up to 95 %, and the experimental results can also achieve 85 %. The experimental results are in good agreement with the simulation, which indicates the feasibility of a chip integrating four channels for two orthogonal polarization base generations. The slight deviation between experimental and simulation results is caused by fabrication error, and the angle between the sample and the incident polarization. The visible noise comes from the system itself.

### 3.2 Four-channel vortex beam generation

**Figure 5:**
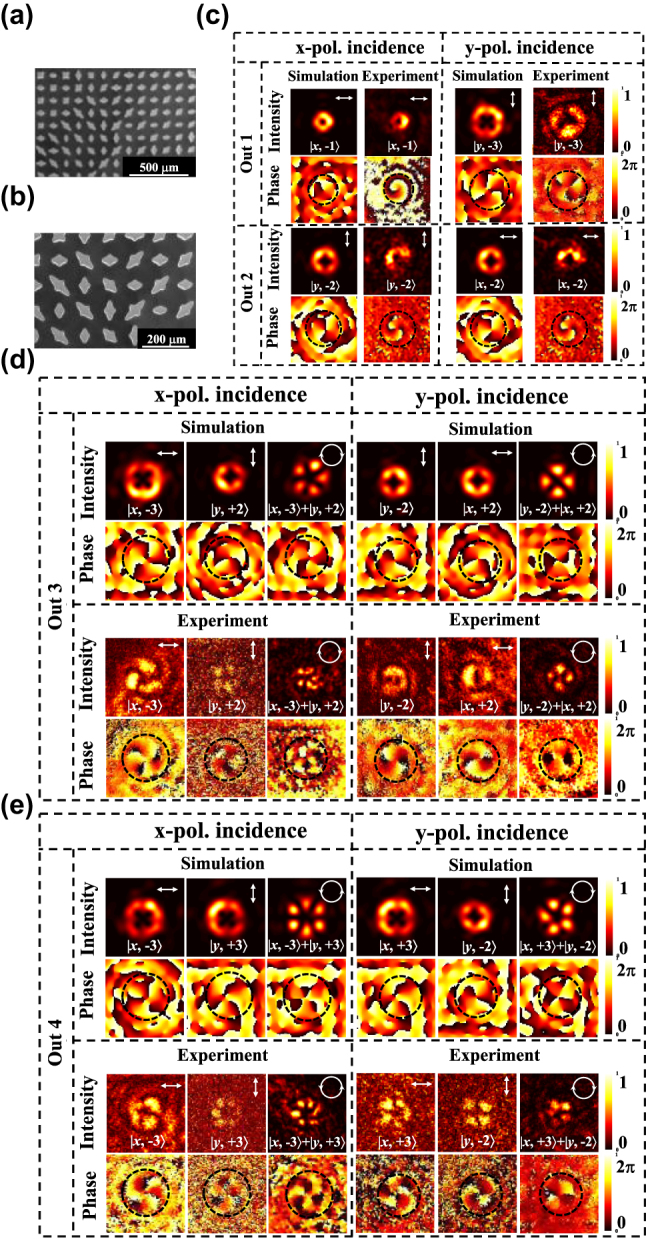
Experimental and simulated results for the generation of orthogonally polarized vortex beams at various topological charges. (a–b) SEM images of the fabricated metasurface. (c–e) The intensity and phase profiles of vortex beam and superposition vortex states under the *x*- or *y*-polarization light incidence, respectively. Fundamental vortex states were generated in channels 1 and 2, and the superposition vortex states were produced in channels 3 and 4.

After demonstrating the feasibility of generating four-channel two orthogonal polarization bases on a single metasurface, we proposed a chip-integrated polarization multiplexed metasurface for simultaneous generation of four-channel orthogonal polarized vortex beams at various topological charges under the orthogonal polarized light incident. Figure 5(a) and (b) is the SEM image of the proposed metasurface. The vortex beam has a helical phase distribution described by exp(*iℓθ*), where *θ* is the azimuthal angle and *ℓ* is the topological charge. Each photon carries an OAM of *ℓℏ* [29]. The intensity profile distribution of the vertex beam with a topological charge of *ℓ* is a “doughnut,” and the beam center has dark points, which is due to the phase singularity. The radius of the “doughnut” defined by the distance from the center to the maximum intensity points is expressed by $r=w\sqrt{l/2}$ , where *w* is the beam radius [28]. The control of polarization and topological charges in vortex beams was realized by properly arranging birefringent meta-atom arrays to induce additional phases of *x*- and *y*-polarization. By control of the phase response of polarization-maintaining and polarization-conversion meta-atoms, the linear co- and cross-polarization of fundamental vortex states were performed in the 1 and 2 channels. The phase distributions are as follows:

$$\;\varphi_{\mathrm{xx}1}=\frac{2\pi }{\lambda }\left(\sqrt{{\left(x-\xi \right)}^{2}+{\left(y-\xi \right)}^{2}+f^{2}}-f\right)-\theta$$

(7) $$\varphi_{\mathrm{yy}1}=\frac{2\pi }{\lambda }\left(\sqrt{{\left(x-\xi \right)}^{2}+{\left(y-\xi \right)}^{2}+f^{2}}-f\right)-3\theta$$

(8) $$\;\varphi_{\text{cross}2}=\frac{2\pi }{\lambda }\left(\sqrt{{\left(x+\xi \right)}^{2}+{\left(y-\xi \right)}^{2}+f^{2}}-f\right)-2\theta$$

where the focal length f is 6,000 µm, the factor is 2,550 μm, and the working frequency is 1.3 THz. From equation (10), we can observe that an *x*-polarized vortex beam at a topological charge of −1 is produced when the *x*-polarization is illuminated. Under *y*-polarization incidence, a *y*-polarized vortex beam at a topological charge of −3 is generated in the first channel. In the second channel, from equation (7), we can find that the generated vortex beam has a topological charge of −2, and its polarization converts to the cross-polarization of the incident when the *x*- or *y*-polarization is incident. As shown in Figure 5(c), by changing the incident from *x*-polarization to *y*-polarization, the produced polarization and topological charge of the vortex beam have changed accordingly in channel 1, and the polarization state has cross-converted in channel 2. The experimental results are in good agreement with the simulation results. The measured intensity distributions have slight uniformity, but the phase distributions are completely identical. This is caused by the fabrication error of deep silicon etching and the low signal-to-noise ratio of the imaging system at 1.3 THz.

The phase and amplitude of the vortex beam have a special spatial distribution in the plane perpendicular to its propagation direction. A coaxial superposition of two vortex beams with certain topological charges and orthogonal circular polarizations can produce an interference pattern beam with a determined number of lobes [34]. The designed metasurface can simultaneously produce cross-polarized and copolarized vortex beams with opposite topological charges in channels 3 or 4. The phase distribution can be described as follows:

$$\;\varphi_{\mathrm{xx}3}=\frac{2\pi }{\lambda }\left(\sqrt{{\left(x+\xi \right)}^{2}+{\left(y+\xi \right)}^{2}+f^{2}}-f\right)-3\theta$$

(9) $$\;\varphi_{\mathrm{yy}3}=\frac{2\pi }{\lambda }\left(\sqrt{{\left(x+\xi \right)}^{2}+{\left(y+\xi \right)}^{2}+f^{2}}-f\right)-2\theta$$

$$\begin{array}{ll} \varphi_{\text{cross}3} & =\frac{2\pi }{\lambda }\left(\sqrt{{\left(x+\xi +\frac{P}{2}\right)}^{2}+{\left(y+\xi -\frac{P}{2}\right)}^{2}+f^{2}}-f\right)\\ & \;+2\theta \end{array}$$

$$\;\varphi_{\mathrm{xx}4}=\frac{2\pi }{\lambda }\left(\sqrt{{\left(x-\xi \right)}^{2}+{\left(y+\xi \right)}^{2}+f^{2}}-f\right)-3\theta$$

(10) $$\varphi_{\mathrm{yy}4}=\frac{2\pi }{\lambda }\left(\sqrt{{\left(x-\xi \right)}^{2}+{\left(y+\xi \right)}^{2}+f^{2}}-f\right)-2\theta$$

$$\begin{array}{ll} \varphi_{\text{cross}4} & =\frac{2\pi }{\lambda }\left(\sqrt{{\left(x-\xi +\frac{P}{2}\right)}^{2}+{\left(y+\xi -\frac{P}{2}\right)}^{2}+f^{2}}-f\right)\\ & \;+3\theta \end{array}$$

In channel 3, it can be observed from Figure 5(d). When an *x*-polarization is incident, an *x*-polarized vortex beam at a topological charge of −3 and the *y*-polarized vortex beam at a topological charge of $+2\left(\left(\left|x,-3\right\rangle +\left|y,+2\right\rangle \right)/\sqrt{2}\right)$ interference obtains a 5-lobed petal-like RCP beam, the number of lobes is equal to the sum of the topological charges of the two interference vortices, and the reason for formatting lobes is due to phase singularity [35]. The interference between an *x*-polarized vortex beam at a topological charge of −2 and a *y*-polarized vortex beam with a topological charge of $+2\left(\left(\left|x,-2\right\rangle +\left|y,+2\right\rangle \right)/\sqrt{2}\right)$ is demonstrated under the *y*-polarization incidence, resulting in a 4-lobed petal-like LCP beam. In channel 4, as shown in Figure 5(e). When the *x*-polarization is incident, a 6-lobed petal-like LCP beam is achieved by the superposition of two vortex beams $\left(\left(\left|x,-3\right\rangle +\left|y,+3\right\rangle \right)/\sqrt{2}\right)$ , the combination of the two vortex beams with $\left(\left(\left|x,+3\right\rangle +\left|y,-2\right\rangle \right)/\sqrt{2}\right)$ gives rise to a 5-lobed petal-like RCP beam, under the *x*-polarization incidence. In the third and fourth channels, the superposition vortex states were generated using orthogonally circularly polarized light. Each vortex’s superposition and circular polarization result from the combination of *x*- and *y*-polarized light components. The distribution of the vortex’s superposition and polarization is determined by both phase and amplitude. Notably, as shown in Figure 2(b), (e), and (f), the transmission amplitude of individual meta-atoms is not constant. However, the total amplitude remains balanced between the copolarized component from all polarization-maintaining meta-atoms and the cross-polarized component from all polarization-conversion meta-atoms in the metasurface design. We can find that the experimental results are coincident with the simulation results and align with the results presented in Ref. [29]. The discrepancies between the experimental and simulation results primarily arise from fabrication errors in the samples, measurement system noise, and system misalignment. Fabrication imperfections in the samples lead to imaging distortions, while system noise reduces imaging contrast. Additionally, misalignment of the measurement system disrupts the precise superposition of orthogonally polarized vortex beams. These factors collectively contribute to the degradation of imaging quality. In channels 1 and 2, a pure vortex beam is generated without any superposition of vortex beams. In contrast, the other channels involve the superposition of orthogonally polarized vortex beams, resulting in complex field distributions. Notably, system misalignment significantly impacts the superposition process of orthogonally polarized vortex beams, causing the experimental results for superimposed vortex beams to be less accurate compared to those of pure vortex beams.

The proposed chip-integrated metasurface can generate four-channel orthogonal polarized vortex beams at various topological charges in the terahertz regime. Simultaneous control of polarization and topological charges in vortex beams shows more degrees of freedom for carrying information. This method effectively overcomes intercoupling effects among the four output channels, enabling independent wavefront shaping for each channel with optimal efficiency. The design supports simultaneous operation with two pairs of orthogonal polarization eigenstates and facilitates the generation of vectorial vortex beams. Unlike conventional metasurfaces [21], [22], [23] that rely on complex phase profiles to achieve multifunctionality, the chip-integrated multisection metalens geometry offers a versatile design platform for customized applications.

## 4 Conclusions

In summary, we presented a chip-integrated polarization multiplexed metasurfaces approach to simultaneously generate orthogonal basis vortices with both linear polarization (*x*- and *y*-polarity) and circular polarization (left- and right-handed polarity) at various predefined topological charges under the orthogonal polarized light incident, showing more degrees of freedom for carrying information. A new method of controlling the phase difference between two fundamental linearly polarized vortices was employed to flexibly manipulate the polarization of OAM superposition states. Experimentally, an all-dielectric metasurface in the terahertz regime was prepared to demonstrate the simultaneous generation of four-channel orthogonal polarized vortex beams at various topological charges. By control of the phase response of polarization-maintaining and polarization-conversion meta-atoms, the linear co- and cross-polarization of fundamental vortex states were performed in the 1 and 2 channels; meanwhile, the RCP and LCP of superposition vortex states were produced in the 3 and 4 channels. Orthogonal linear and circular polarization vortex beams, as well as various topological charges, were measured in four spatial-decoupling channels. The experimental results are in good agreement with the simulations. Such a metasurface approach provides complete polarization bases for further synthesis of diverse polarization vortices required for huge-capacity communication.

## Supplementary Material

Supplementary Material Details

## References

[1] Willner A. A. E. (2015). Optical communications using orbital angular momentum beams. *Adv. Opt. Photonics*.

[2] Wang J. (2012). Terabit free-space data transmission employing orbital angular momentum multiplexing. *Nat. Photonics*.

[3] Lei T. (2015). Massive individual orbital angular momentum channels for multiplexing enabled by Dammann gratings. *Light: Sci. Appl.*.

[4] Gahagan K. T., Swartzlander G. A. (1996). Optical vortex trapping of particles. *Opt. Lett.*.

[5] Padgett M., Bowman R. (2011). Tweezers with a twist. *Nat. Photonics*.

[6] Nicolas A., Veissier L., Giner L., Giacobino E., Maxein D., Laurat J. (2014). A quantum memory for orbital angular momentum photonic qubits. *Nat. Photonics*.

[7] Bouchard F., Fickler R., Boyd R., Karimi E. (2017). High-dimensional quantum cloning and applications to quantum hacking. *Sci. Adv.*.

[8] Marrucci L. (2011). Spin-to-orbital conversion of the angular momentum of light and its classical and quantum applications. *J. Opt.*.

[9] Zhou Z. Y. (2022). Time-varying orbital angular momentum in tight focusing of ultrafast pulses. *Opt. Express*.

[10] Andersen M. F. (2006). Quantized rotation of atoms from photons with orbital angular momentum. *Phys. Rev. Lett.*.

[11] Vaziri A., Weihs G., Zeilinger A. (2002). Superpositions of the orbital angular momentum for applications in quantum experiments. *J. Opt. B: Quantum Semiclassical Opt.*.

[12] Berg-Johansen S. (2015). Classically entangled optical beams for high-speed kinematic sensing. *Optica*.

[13] Lavery M. P. J., Speirits F. C., Barnett S. M., Padgett M. J. (2013). Detection of a spinning object using light’s orbital angular momentum. *Science*.

[14] Beijersbergen M. W., Coerwinkel R. P. C., Kristensen M., Woerdman J. P. (1994). Helical-wavefront laser beams produced with a spiral phaseplate. *Opt. Commun.*.

[15] Beijersbergen M. W., Allen L., van der Veen H. E. L. O., Woerdman J. P. (1993). Astigmatic laser mode converters and transfer of orbital angular momentum. *Opt. Commun.*.

[16] Guo C. S., Liu X., Ren X. Y., Wang H. T. (2005). Optimal annular computer-generated holograms for the generation of optical vortices. *J. Opt. Soc. Am. A*.

[17] Fickler R., Krenn M., Lapkiewicz R., Ramelow S., Zeilinger A. (2013). Real-time imaging of quantum entanglement. *Sci. Rep.*.

[18] Yu N. (2011). Light propagation with phase discontinuities: generalized laws of reflection and refraction. *Science*.

[19] Qin F. (2016). Hybrid bilayer plasmonic metasurface efficiently manipulates visible light. *Sci. Adv.*.

[20] Liu L. X. (2022). Spatial coherence manipulation on the disorder-engineered statistical photonic platform. *Nano Lett.*.

[21] Zang X. F. (2019). A multi-foci metalens with polarization-rotated focal points. *Laser Photonics Rev.*.

[22] Wang R. X. (2023). Compact multi-foci metalens spectrometer. *Light Sci. Appl.*.

[23] Wang R. X. (2021). Metalens for generating a customized vectorial focal curve. *Nano Lett.*.

[24] Zhu Y., Zang X. F., Chi H. X., Zhou Y. W., Zhu Y. M., Zhuang S. L. (2023). Metasurfaces designed by a bidirectional deep neural network and iterative algorithm for generating quantitative field distributions. *Light Adv. Manuf.*.

[25] Xia S. X., Zhang D., Zhai X., Wang L. L., Wen S. C. (2023). Phase-controlled topological plasmons in 1D graphene nanoribbon array. *Appl. Phys. Lett.*.

[26] Chi H. X. (2025). Metasurface enabled multi-target and multi-wavelength diffraction neural networks. *Laser Photonics Rev.*.

[27] Zhou Y. W. (2024). Directional phase and polarization manipulation using Janus metasurfaces. *Adv. Sci.*.

[28] Wang Q. (2018). Reflective chiral meta-holography: multiplexing holograms for circularly polarized waves. *Light Sci. Appl.*.

[29] Deng Z. L. (2021). Vectorial compound metapixels for arbitrary nonorthogonal polarization steganography. *Adv. Mater.*.

[30] Li J. T. (2022). Dynamic phase assembled terahertz metalens for reversible conversion between linear polarization and arbitrary circular polarization. *Opto-Electron. Adv.*.

[31] Yue Z. (2023). All-dielectric terahertz metasurfaces with dual-functional polarization manipulation for orthogonal polarization states. *Nanoscale*.

[32] Zhang Q. Y. (2021). Spin decoupling metasurfaces for four-channel terahertz wavefront shaping. *Adv. Opt. Mater.*.

[33] Li J. (2021). All-silicon metasurfaces for polarization multiplexed generation of terahertz photonic orbital angular momentum superposition states. *J. Mater. Chem. C*.

[34] Li F. Y. (2023). All-dielectric terahertz metasurface for linearly-polarized multichannel transmission and superposition states of spherical and vortex waves. *Photonics Res.*.

[35] Zheng C. L. (2021). All-dielectric metasurface for manipulating the superpositions of orbital angular momentum via spin-decoupling. *Adv. Opt. Mater.*.

[36] Yue F. Y. (2017). Multichannel polarization-controllable superpositions of orbital angular momentum states. *Adv. Mater.*.

[37] Zhang Z. Y. (2020). Coherent perfect diffraction in metagratings. *Adv. Mater.*.

