# Supplementary material for: Chip-integrated polarization multiplexed metasurface for simultaneous generation of versatile terahertz vortices
Source: Nanophotonics. 2025 Apr 22;14(12):2219–28. doi: 10.1515/nanoph-2025-0091 (PMC12147553; doi:10.1515/nanoph-2025-0091)
Supplement: Supplementary file 1 — Supplementary Material Details [file j_nanoph-2025-0091_suppl_001.docx]

Supporting Information

**Chip-integrated** **polarization** **multiplexed metasurface for simultaneous generation of versatile terahertz vortices**

Qianyun Zhang, Guibin Li, Liang Wu*, Fan Yang, Zhen Yue, Chenglong Zheng, Yan Zhang*, Li Li*, and Jianquan Yao*

Q. Zhang, L. Wu, F. Yang, C. Zheng, J. Yao

Key Laboratory of Optoelectronics Information and Technology of Ministry of Education

College of Precision Instruments and Opto-Electronics Engineering

Tianjin University

No. 92 WeiJin Road, Tianjin 300072, China

G. Li, Y. Zhang

Beijing Key Laboratory for Metamaterials and Devices,

Key Laboratory of Terahertz Optoelectronics of Ministry of Education,

and Beijing Advanced Innovation Center for Imaging Technology

Department of Physics

Capital Normal University

Beijing 100048, China

Z. Yue

Department of Optoelectronic Information Science and Engineering,

Jiangsu University

Zhenjiang 212013, China

L. Li

College of Physics

Harbin Institute of Technology

Harbin 150000, China

Corresponding authors: wuliang@tju.edu.cn; [yzhang@mail.cnu.edu.cn](mailto:yzhang@mail.cnu.edu.cn)；[lili.phys@hit.edu.cn](mailto:lili.phys@hit.edu.cn)；jqyao@tju.edu.cn

Section S1: The design of meta-atoms

Under the x- and y-polarization incidence, the transmission amplitude and phase shift of the two meta-atoms are shown in Figure S1. The working frequency is 1.3 THz. Fig. S1(a-d) corresponds to the unit (Ⅰ), where T_xx_, T_yy_, φ_xx_, and φ_yy_ represent the co-polarization transmission amplitude and phase shift for the x- and y-polarized incident lights. As seen in Fig. S1(a) and (b), the values of T_xx_ and T_yy_ are almost above 0.6 (given that the reflection loss of high-resistance silicon is approximately 30%), meeting the intensity requirements for the transmission field. In Fig. S1(c) and (d), the phase shift covers 360 degrees within the scanning range, providing flexible phase manipulation. For unit (Ⅱ), when the x-polarized light is incident, the transmission amplitude and phase value of the y-polarized field are equal to that of the x-polarized field when the y-polarized light is incident. Therefore, we use a uniform symbol (T_cross_ and φ_cross_) to represent them. As shown in Fig. S1(e) and (f), the amplitude is also approximately around 0.6, and the phase shift covers more than 180 degrees. When the meta-atom values of Lx2 and Ly2 are exchanged to rotate the structure by 90 degrees, the phase delay compared to the unexchanged structure increases by 180 degrees, resulting in a total phase delay of 360 degrees. A parameter library for the two types of units has been established.

Section S2: Sample Fabrication

We used standard ultraviolet lithography and inductively coupled plasma (ICP) etching to fabricate the proposed versatile polarization states generation metasurface. The positive photoresist (AZ4620) was spin-coated on a 500 μm thick, clean silicon wafer with a diameter of 4 inches, and formed a 6.8 μm thick photoresist film pattern by prebaking as a mask. The mask was aligned with the silicon wafer, followed by exposure and development. After that, the etching technology (STS MULTIPLEX ASE-HRMICP ETCHER, United Kingdom) was used to etch the sample. Finally, the remaining photoresist was removed, and the final sample was obtained.

**Section S3: THz holographic imaging system**

The intensity and phase distributions of the transmitted field were measured using a THz holographic imaging system. The light emitted from a femtosecond laser amplifier system (pulse width of 50 fs, working wavelength of 800 nm, repetition frequency of 1 kHz) is divided into a pump beam and a probe beam. The pump beam irradiates the electro-optic crystal ZnTe to produce terahertz waves based on the optical rectification effect. The generated terahertz wave is collimated by an off-axis parabolic mirror and illuminated onto the sample. A half-wave plate (HWP) and a polarizer (P) were used to manipulate the polarization states of the probe beam. The terahertz wave passing through the sample overlaps with the probe beam in the detection ZnTe crystal. In the detection of ZnTe crystal, due to the Pockels effect, the polarization state of the probe beam is modulated by the terahertz electric field. The modulated probe beam is then reflected by the detection crystal and directed to the imaging system. The optical imaging module consists of a quarter-wave plate (QWP), a Wollaston prism (WP), two lenses, and a charge-coupled device (CCD). The THz field information was obtained using balanced electro-optic detection technique and the dynamic subtraction technique.

**Figure S1**. The transmission amplitudes and phase shifts of the two geometric meta-atoms with the function of Lx and Ly.
